# *N*-{2-[4-(2-Meth­oxy­phen­yl)piperazin-1-yl]eth­yl}-4-nitro-*N*-(2-pyrid­yl)benzamide

**DOI:** 10.1107/S1600536810038080

**Published:** 2010-09-30

**Authors:** Chunxiong Lu, Quanfu Jiang

**Affiliations:** a Key Laboratory of Nuclear Medicine, Ministry of Health, Jiangsu Key Laboratory of Molecular Nuclear Medicine, Jiangsu Institute of Nuclear Medicine, Wuxi 214063, People’s Republic of China

## Abstract

In the title compound, C_25_H_27_N_5_O_4_, the piperizine ring adopts a chair conformation. The dihedral angles between the pyridine ring and the two benzene rings are 65.5 (4) and 70.7 (4)°, while the dihedral angle between the two benzene rings is 17.3 (3)°. An intra­molecular C—H⋯O hydrogen bond occurs.

## Related literature

For the use of the title compound as a labeling precursor of the serotonin (5-HT_1 A_) receptor imaging agent,^18^F-MPPF, see: Le Bars *et al.* (1998 ▶); Zhuang *et al.* (1994 ▶).
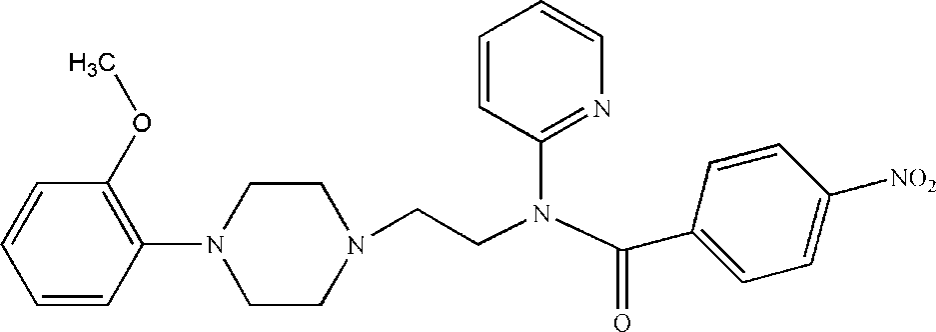


## Experimental

### 

#### Crystal data

C_25_H_27_N_5_O_4_
*M* *_r_* = 461.52
Monoclinic, 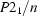

*a* = 11.480 (2) Å
*b* = 15.512 (3) Å
*c* = 13.235 (2) Å
β = 108.505 (3)°
*V* = 2234.9 (7) Å^3^
*Z* = 4
Mo *K*α radiation
μ = 0.10 mm^−1^
*T* = 103 K
0.50 × 0.50 × 0.33 mm

#### Data collection

Rigaku SPIDER diffractometer
17307 measured reflections
5066 independent reflections
4318 reflections with *I* > 2σ(*I*)
*R*_int_ = 0.028

#### Refinement

*R*[*F* ^2^ > 2σ(*F* ^2^)] = 0.041
*wR*(*F* ^2^) = 0.098
*S* = 1.00
5066 reflections
308 parameters
H-atom parameters constrained
Δρ_max_ = 0.31 e Å^−3^
Δρ_min_ = −0.17 e Å^−3^

### 

Data collection: *RAPID-AUTO* (Rigaku, 2004 ▶); cell refinement: *RAPID-AUTO*; data reduction: *RAPID-AUTO*; program(s) used to solve structure: *SHELXS97* (Sheldrick, 2008 ▶); program(s) used to refine structure: *SHELXL97* (Sheldrick, 2008 ▶); molecular graphics: *SHELXTL* (Sheldrick, 2008 ▶); software used to prepare material for publication: *SHELXTL*.

## Supplementary Material

Crystal structure: contains datablocks I, global. DOI: 10.1107/S1600536810038080/fk2025sup1.cif

Structure factors: contains datablocks I. DOI: 10.1107/S1600536810038080/fk2025Isup2.hkl

Additional supplementary materials:  crystallographic information; 3D view; checkCIF report

## Figures and Tables

**Table 1:** Hydrogen-bond geometry (Å, °)

*D*—H⋯*A*	*D*—H	H⋯*A*	*D*⋯*A*	*D*—H⋯*A*
C10—H10*A*⋯O1	0.99	2.35	2.9536 (18)	119

**Fig. 1.**
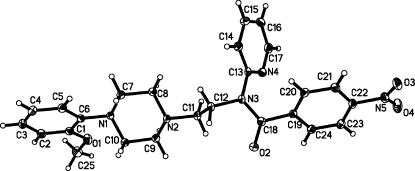
The molecular structure of the title compound, with the atomic numbering scheme. Displacement ellipsoids are drawn at the 50% probability level.

## supplementary crystallographic information

### Comment

*N*-{2-[4-(2-Methoxyphenyl)piperazin-1-yl]ethyl}-4-nitro-*N*-(2-pyridyl)benzamide, (I), is a labeling precursor of 18F-MPPF, serotonin (5-HT1A) receptor imaging agents (18F-MPPF = 4-(2-methoxyphenyl)-1-[2-(*N*-2-pyridinyl)-*p*-18F-fluorobenzamido]ethylpiperazine). We report here the crystal structure of (I). Two phenyl rings and pyridine rings are planar with a maximum deviation of 0.004 (2)Å for atom C1, 0.010 (9)Å for atom C20 and 0.012 (7)Å for atom N4. The dihedral angles between the pyridine ring and the other two phenyl rings are 65.5 (4) and 70.7 (4)°, respectively, while the dihedral angle between the two phenyl rings is 17.3 (3)°. The piperazine ring adopts a chair conformation. Intramolecular C—H···O hydrogen bond could be found in the crystal structure of the title compound.

### Experimental

The title compound was synthesized according to the method reported in the literature(Zhuang *et al.*,1994) and crystallized from a mixed solvent composed of ethyl acetate and petroleum ether (1:1); colorless block-shaped crystals were obtained after several days.

### Refinement

Positional parameters of all the H atoms bonded to C atoms were calculated geometrically and were allowed to ride on the C atoms to which they are attached with C—H distances of 0.95Å (CH), 0.98Å (CH_3_) or 0.99Å (CH_2_), and with *U*_iso_(H) = 1.2U_eq_ (or 1.5 for CH_3_) of the parent atoms.

### Crystal data

**Table:**

C_25_H_27_N_5_O_4_	*F*(000) = 976
*M**_r_* = 461.52	*D*_x_ = 1.372 Mg m^−^^3^
Monoclinic, *P*2_1_/*n*	Mo *K*α radiation, λ = 0.71073 Å
Hall symbol: -P 2yn	Cell parameters from 6374 reflections
*a* = 11.480 (2) Å	θ = 3.1–27.5°
*b* = 15.512 (3) Å	µ = 0.10 mm^−^^1^
*c* = 13.235 (2) Å	*T* = 103 K
β = 108.505 (3)°	Block, yellow
*V* = 2234.9 (7) Å^3^	0.50 × 0.50 × 0.33 mm
*Z* = 4	

### Data collection

**Table:**

Rigaku SPIDER diffractometer	4318 reflections with *I* > 2σ(*I*)
Radiation source: Rotating Anode	*R*_int_ = 0.028
graphite	θ_max_ = 27.5°, θ_min_ = 3.1°
ω scans	*h* = −14→14
17307 measured reflections	*k* = −20→19
5066 independent reflections	*l* = −13→17

### Refinement

**Table:**

Refinement on *F*^2^	Primary atom site location: structure-invariant direct methods
Least-squares matrix: full	Secondary atom site location: difference Fourier map
*R*[*F*^2^ > 2σ(*F*^2^)] = 0.041	Hydrogen site location: inferred from neighbouring sites
*wR*(*F*^2^) = 0.098	H-atom parameters constrained
*S* = 1.00	*w* = 1/[σ^2^(*F*_o_^2^) + (0.0465*P*)^2^ + 0.850*P*] where *P* = (*F*_o_^2^ + 2*F*_c_^2^)/3
5066 reflections	(Δ/σ)_max_ = 0.001
308 parameters	Δρ_max_ = 0.31 e Å^−^^3^
0 restraints	Δρ_min_ = −0.17 e Å^−^^3^

### Special details

**Table:**

Geometry. All e.s.d.'s (except the e.s.d. in the dihedral angle between two l.s. planes) are estimated using the full covariance matrix. The cell e.s.d.'s are taken into account individually in the estimation of e.s.d.'s in distances, angles and torsion angles; correlations between e.s.d.'s in cell parameters are only used when they are defined by crystal symmetry. An approximate (isotropic) treatment of cell e.s.d.'s is used for estimating e.s.d.'s involving l.s. planes.
Refinement. Refinement of *F*^2^ against ALL reflections. The weighted *R*-factor *wR* and goodness of fit *S* are based on *F*^2^, conventional *R*-factors *R* are based on *F*, with *F* set to zero for negative *F*^2^. The threshold expression of *F*^2^ > σ(*F*^2^) is used only for calculating *R*-factors(gt) *etc*. and is not relevant to the choice of reflections for refinement. *R*-factors based on *F*^2^ are statistically about twice as large as those based on *F*, and *R*- factors based on ALL data will be even larger.

### Fractional atomic coordinates and isotropic or equivalent isotropic displacement parameters (Å^2^)

**Table:**

	*x*	*y*	*z*	*U*_iso_*/*U*_eq_	
O1	0.63130 (9)	0.66210 (6)	0.68937 (8)	0.0225 (2)	
O2	0.27919 (9)	0.13878 (6)	0.52481 (7)	0.0206 (2)	
O3	0.25952 (9)	−0.31358 (6)	0.59155 (8)	0.0251 (2)	
O4	0.35176 (10)	−0.30235 (6)	0.47275 (9)	0.0291 (2)	
N1	0.53263 (10)	0.53783 (7)	0.77943 (8)	0.0151 (2)	
N2	0.44755 (10)	0.36714 (6)	0.70911 (8)	0.0158 (2)	
N3	0.39572 (10)	0.12774 (6)	0.69878 (8)	0.0149 (2)	
N4	0.57867 (10)	0.05037 (7)	0.73066 (9)	0.0171 (2)	
N5	0.30875 (10)	−0.27110 (7)	0.53821 (9)	0.0192 (2)	
C1	0.57913 (11)	0.68987 (8)	0.76307 (10)	0.0171 (3)	
C2	0.57713 (12)	0.77550 (8)	0.79298 (11)	0.0211 (3)	
H2	0.6113	0.8187	0.7600	0.025*	
C3	0.52534 (13)	0.79824 (9)	0.87100 (11)	0.0227 (3)	
H3	0.5244	0.8569	0.8913	0.027*	
C4	0.47553 (13)	0.73621 (9)	0.91885 (11)	0.0220 (3)	
H4	0.4405	0.7518	0.9724	0.026*	
C5	0.47655 (12)	0.64994 (9)	0.88842 (10)	0.0187 (3)	
H5	0.4417	0.6073	0.9217	0.022*	
C6	0.52736 (11)	0.62517 (8)	0.81082 (10)	0.0158 (3)	
C7	0.51121 (12)	0.47400 (8)	0.85260 (10)	0.0175 (3)	
H7A	0.4262	0.4796	0.8549	0.021*	
H7B	0.5683	0.4844	0.9253	0.021*	
C8	0.53066 (13)	0.38377 (8)	0.81686 (10)	0.0194 (3)	
H8A	0.6168	0.3773	0.8179	0.023*	
H8B	0.5155	0.3411	0.8669	0.023*	
C9	0.47539 (12)	0.42937 (8)	0.63708 (10)	0.0177 (3)	
H9A	0.4223	0.4183	0.5631	0.021*	
H9B	0.5620	0.4229	0.6396	0.021*	
C10	0.45397 (12)	0.52014 (8)	0.66945 (10)	0.0176 (3)	
H10A	0.4726	0.5620	0.6202	0.021*	
H10B	0.3666	0.5271	0.6645	0.021*	
C11	0.45819 (12)	0.27806 (8)	0.67654 (10)	0.0170 (3)	
H11A	0.5445	0.2587	0.7063	0.020*	
H11B	0.4342	0.2748	0.5979	0.020*	
C12	0.37576 (12)	0.21947 (8)	0.71606 (10)	0.0163 (3)	
H12A	0.3916	0.2297	0.7931	0.020*	
H12B	0.2888	0.2342	0.6785	0.020*	
C13	0.49869 (11)	0.08612 (8)	0.77229 (10)	0.0144 (2)	
C14	0.51295 (12)	0.08617 (8)	0.88043 (10)	0.0187 (3)	
H14	0.4544	0.1134	0.9069	0.022*	
C15	0.61517 (13)	0.04521 (9)	0.94863 (11)	0.0222 (3)	
H15	0.6285	0.0446	1.0232	0.027*	
C16	0.69744 (13)	0.00529 (9)	0.90684 (11)	0.0212 (3)	
H16	0.7670	−0.0245	0.9517	0.025*	
C17	0.67581 (12)	0.00988 (9)	0.79809 (11)	0.0206 (3)	
H17	0.7328	−0.0171	0.7695	0.025*	
C18	0.33804 (11)	0.09378 (8)	0.59942 (10)	0.0150 (2)	
C19	0.33808 (11)	−0.00235 (8)	0.58734 (10)	0.0145 (3)	
C20	0.31415 (12)	−0.05593 (8)	0.66290 (10)	0.0163 (3)	
H20	0.3063	−0.0319	0.7265	0.020*	
C21	0.30169 (12)	−0.14415 (8)	0.64579 (10)	0.0178 (3)	
H21	0.2827	−0.1809	0.6958	0.021*	
C22	0.31762 (11)	−0.17711 (8)	0.55421 (10)	0.0163 (3)	
C23	0.34266 (12)	−0.12582 (8)	0.47817 (10)	0.0177 (3)	
H23	0.3541	−0.1504	0.4163	0.021*	
C24	0.35056 (12)	−0.03751 (8)	0.49471 (10)	0.0173 (3)	
H24	0.3646	−0.0007	0.4424	0.021*	
C25	0.68011 (14)	0.72601 (9)	0.63702 (12)	0.0257 (3)	
H25A	0.6140	0.7646	0.5969	0.039*	
H25B	0.7169	0.6981	0.5880	0.039*	
H25C	0.7430	0.7593	0.6901	0.039*	

### Atomic displacement parameters (Å^2^)

**Table:**

	*U*^11^	*U*^22^	*U*^33^	*U*^12^	*U*^13^	*U*^23^
O1	0.0285 (5)	0.0173 (5)	0.0263 (5)	−0.0025 (4)	0.0150 (4)	0.0013 (4)
O2	0.0239 (5)	0.0177 (5)	0.0172 (5)	0.0010 (4)	0.0020 (4)	0.0021 (4)
O3	0.0281 (5)	0.0165 (5)	0.0313 (6)	−0.0037 (4)	0.0104 (4)	0.0022 (4)
O4	0.0346 (6)	0.0198 (5)	0.0385 (6)	0.0003 (4)	0.0194 (5)	−0.0083 (4)
N1	0.0191 (5)	0.0111 (5)	0.0137 (5)	−0.0013 (4)	0.0031 (4)	0.0002 (4)
N2	0.0216 (6)	0.0110 (5)	0.0136 (5)	−0.0013 (4)	0.0036 (4)	−0.0009 (4)
N3	0.0176 (5)	0.0110 (5)	0.0154 (5)	0.0004 (4)	0.0041 (4)	−0.0016 (4)
N4	0.0165 (5)	0.0167 (5)	0.0176 (5)	−0.0006 (4)	0.0048 (4)	0.0002 (4)
N5	0.0176 (5)	0.0145 (5)	0.0244 (6)	0.0003 (4)	0.0050 (5)	−0.0013 (4)
C1	0.0161 (6)	0.0169 (6)	0.0164 (6)	−0.0007 (5)	0.0022 (5)	0.0003 (5)
C2	0.0236 (7)	0.0148 (6)	0.0218 (7)	−0.0027 (5)	0.0027 (5)	0.0017 (5)
C3	0.0271 (7)	0.0148 (6)	0.0223 (7)	−0.0007 (5)	0.0022 (6)	−0.0034 (5)
C4	0.0250 (7)	0.0208 (7)	0.0184 (7)	0.0013 (5)	0.0043 (5)	−0.0037 (5)
C5	0.0201 (6)	0.0173 (6)	0.0174 (6)	−0.0013 (5)	0.0042 (5)	0.0000 (5)
C6	0.0152 (6)	0.0131 (6)	0.0159 (6)	−0.0005 (5)	0.0003 (5)	−0.0001 (4)
C7	0.0226 (6)	0.0139 (6)	0.0150 (6)	−0.0024 (5)	0.0045 (5)	−0.0001 (5)
C8	0.0258 (7)	0.0139 (6)	0.0153 (6)	−0.0011 (5)	0.0018 (5)	0.0011 (5)
C9	0.0234 (7)	0.0146 (6)	0.0143 (6)	−0.0011 (5)	0.0047 (5)	−0.0001 (5)
C10	0.0206 (6)	0.0141 (6)	0.0154 (6)	−0.0002 (5)	0.0018 (5)	0.0011 (5)
C11	0.0211 (6)	0.0130 (6)	0.0163 (6)	0.0004 (5)	0.0049 (5)	−0.0013 (5)
C12	0.0194 (6)	0.0117 (6)	0.0175 (6)	0.0016 (5)	0.0051 (5)	−0.0020 (5)
C13	0.0160 (6)	0.0100 (5)	0.0162 (6)	−0.0028 (5)	0.0036 (5)	−0.0010 (4)
C14	0.0225 (6)	0.0175 (6)	0.0169 (6)	−0.0005 (5)	0.0072 (5)	−0.0004 (5)
C15	0.0285 (7)	0.0212 (7)	0.0145 (6)	−0.0022 (6)	0.0036 (5)	0.0008 (5)
C16	0.0201 (6)	0.0164 (6)	0.0226 (7)	−0.0002 (5)	0.0006 (5)	0.0031 (5)
C17	0.0176 (6)	0.0184 (6)	0.0244 (7)	0.0010 (5)	0.0044 (5)	−0.0020 (5)
C18	0.0149 (6)	0.0150 (6)	0.0157 (6)	−0.0009 (5)	0.0055 (5)	−0.0002 (5)
C19	0.0119 (6)	0.0140 (6)	0.0160 (6)	−0.0007 (5)	0.0019 (5)	−0.0012 (5)
C20	0.0175 (6)	0.0175 (6)	0.0141 (6)	−0.0017 (5)	0.0052 (5)	−0.0021 (5)
C21	0.0178 (6)	0.0171 (6)	0.0183 (6)	−0.0029 (5)	0.0054 (5)	0.0020 (5)
C22	0.0142 (6)	0.0126 (6)	0.0208 (6)	−0.0012 (5)	0.0035 (5)	−0.0019 (5)
C23	0.0187 (6)	0.0183 (6)	0.0166 (6)	−0.0015 (5)	0.0064 (5)	−0.0043 (5)
C24	0.0193 (6)	0.0173 (6)	0.0152 (6)	−0.0022 (5)	0.0053 (5)	0.0008 (5)
C25	0.0288 (7)	0.0225 (7)	0.0285 (8)	−0.0060 (6)	0.0128 (6)	0.0041 (6)

### Geometric parameters (Å, °)

**Table:**

O1—C1	1.3662 (16)	C9—C10	1.5145 (18)
O1—C25	1.4223 (16)	C9—H9A	0.9900
O2—C18	1.2228 (15)	C9—H9B	0.9900
O3—N5	1.2268 (15)	C10—H10A	0.9900
O4—N5	1.2251 (15)	C10—H10B	0.9900
N1—C6	1.4240 (16)	C11—C12	1.5196 (18)
N1—C7	1.4594 (16)	C11—H11A	0.9900
N1—C10	1.4747 (16)	C11—H11B	0.9900
N2—C9	1.4616 (16)	C12—H12A	0.9900
N2—C11	1.4641 (16)	C12—H12B	0.9900
N2—C8	1.4652 (16)	C13—C14	1.3879 (18)
N3—C18	1.3758 (16)	C14—C15	1.3867 (19)
N3—C13	1.4250 (15)	C14—H14	0.9500
N3—C12	1.4705 (15)	C15—C16	1.383 (2)
N4—C13	1.3323 (16)	C15—H15	0.9500
N4—C17	1.3426 (17)	C16—C17	1.383 (2)
N5—C22	1.4722 (16)	C16—H16	0.9500
C1—C2	1.3882 (18)	C17—H17	0.9500
C1—C6	1.4137 (18)	C18—C19	1.4997 (17)
C2—C3	1.391 (2)	C19—C24	1.3901 (18)
C2—H2	0.9500	C19—C20	1.3931 (18)
C3—C4	1.372 (2)	C20—C21	1.3870 (18)
C3—H3	0.9500	C20—H20	0.9500
C4—C5	1.3987 (19)	C21—C22	1.3801 (19)
C4—H4	0.9500	C21—H21	0.9500
C5—C6	1.3866 (19)	C22—C23	1.3828 (18)
C5—H5	0.9500	C23—C24	1.3856 (18)
C7—C8	1.5164 (18)	C23—H23	0.9500
C7—H7A	0.9900	C24—H24	0.9500
C7—H7B	0.9900	C25—H25A	0.9800
C8—H8A	0.9900	C25—H25B	0.9800
C8—H8B	0.9900	C25—H25C	0.9800
			
C1—O1—C25	117.24 (11)	H10A—C10—H10B	108.1
C6—N1—C7	114.85 (10)	N2—C11—C12	110.12 (10)
C6—N1—C10	113.30 (10)	N2—C11—H11A	109.6
C7—N1—C10	110.45 (10)	C12—C11—H11A	109.6
C9—N2—C11	112.05 (10)	N2—C11—H11B	109.6
C9—N2—C8	107.99 (10)	C12—C11—H11B	109.6
C11—N2—C8	111.31 (10)	H11A—C11—H11B	108.2
C18—N3—C13	121.49 (10)	N3—C12—C11	112.36 (10)
C18—N3—C12	117.89 (10)	N3—C12—H12A	109.1
C13—N3—C12	117.95 (10)	C11—C12—H12A	109.1
C13—N4—C17	117.12 (11)	N3—C12—H12B	109.1
O4—N5—O3	123.85 (11)	C11—C12—H12B	109.1
O4—N5—C22	117.94 (11)	H12A—C12—H12B	107.9
O3—N5—C22	118.20 (11)	N4—C13—C14	123.74 (12)
O1—C1—C2	123.77 (12)	N4—C13—N3	115.94 (11)
O1—C1—C6	115.92 (11)	C14—C13—N3	120.30 (11)
C2—C1—C6	120.30 (12)	C15—C14—C13	118.00 (12)
C1—C2—C3	120.27 (13)	C15—C14—H14	121.0
C1—C2—H2	119.9	C13—C14—H14	121.0
C3—C2—H2	119.9	C16—C15—C14	119.28 (13)
C4—C3—C2	120.24 (13)	C16—C15—H15	120.4
C4—C3—H3	119.9	C14—C15—H15	120.4
C2—C3—H3	119.9	C17—C16—C15	118.25 (12)
C3—C4—C5	119.68 (13)	C17—C16—H16	120.9
C3—C4—H4	120.2	C15—C16—H16	120.9
C5—C4—H4	120.2	N4—C17—C16	123.57 (13)
C6—C5—C4	121.48 (13)	N4—C17—H17	118.2
C6—C5—H5	119.3	C16—C17—H17	118.2
C4—C5—H5	119.3	O2—C18—N3	121.88 (11)
C5—C6—C1	118.02 (12)	O2—C18—C19	120.02 (11)
C5—C6—N1	123.12 (11)	N3—C18—C19	117.80 (11)
C1—C6—N1	118.83 (11)	C24—C19—C20	119.87 (12)
N1—C7—C8	110.29 (11)	C24—C19—C18	119.19 (11)
N1—C7—H7A	109.6	C20—C19—C18	120.66 (11)
C8—C7—H7A	109.6	C21—C20—C19	120.34 (12)
N1—C7—H7B	109.6	C21—C20—H20	119.8
C8—C7—H7B	109.6	C19—C20—H20	119.8
H7A—C7—H7B	108.1	C22—C21—C20	118.25 (12)
N2—C8—C7	110.57 (10)	C22—C21—H21	120.9
N2—C8—H8A	109.5	C20—C21—H21	120.9
C7—C8—H8A	109.5	C21—C22—C23	122.82 (12)
N2—C8—H8B	109.5	C21—C22—N5	118.09 (11)
C7—C8—H8B	109.5	C23—C22—N5	119.08 (11)
H8A—C8—H8B	108.1	C22—C23—C24	118.18 (12)
N2—C9—C10	109.93 (10)	C22—C23—H23	120.9
N2—C9—H9A	109.7	C24—C23—H23	120.9
C10—C9—H9A	109.7	C23—C24—C19	120.48 (12)
N2—C9—H9B	109.7	C23—C24—H24	119.8
C10—C9—H9B	109.7	C19—C24—H24	119.8
H9A—C9—H9B	108.2	O1—C25—H25A	109.5
N1—C10—C9	110.41 (10)	O1—C25—H25B	109.5
N1—C10—H10A	109.6	H25A—C25—H25B	109.5
C9—C10—H10A	109.6	O1—C25—H25C	109.5
N1—C10—H10B	109.6	H25A—C25—H25C	109.5
C9—C10—H10B	109.6	H25B—C25—H25C	109.5
			
C25—O1—C1—C2	−3.46 (18)	C17—N4—C13—N3	−179.26 (11)
C25—O1—C1—C6	177.71 (11)	C18—N3—C13—N4	38.82 (16)
O1—C1—C2—C3	−178.09 (12)	C12—N3—C13—N4	−122.24 (12)
C6—C1—C2—C3	0.69 (19)	C18—N3—C13—C14	−142.72 (12)
C1—C2—C3—C4	−0.2 (2)	C12—N3—C13—C14	56.22 (16)
C2—C3—C4—C5	−0.3 (2)	N4—C13—C14—C15	−1.27 (19)
C3—C4—C5—C6	0.2 (2)	N3—C13—C14—C15	−179.60 (11)
C4—C5—C6—C1	0.29 (19)	C13—C14—C15—C16	−0.83 (19)
C4—C5—C6—N1	178.53 (12)	C14—C15—C16—C17	1.7 (2)
O1—C1—C6—C5	178.13 (11)	C13—N4—C17—C16	−1.38 (19)
C2—C1—C6—C5	−0.74 (18)	C15—C16—C17—N4	−0.6 (2)
O1—C1—C6—N1	−0.19 (16)	C13—N3—C18—O2	−155.72 (12)
C2—C1—C6—N1	−179.06 (11)	C12—N3—C18—O2	5.36 (18)
C7—N1—C6—C5	−15.52 (17)	C13—N3—C18—C19	30.55 (17)
C10—N1—C6—C5	112.69 (14)	C12—N3—C18—C19	−168.37 (10)
C7—N1—C6—C1	162.70 (11)	O2—C18—C19—C24	43.64 (17)
C10—N1—C6—C1	−69.08 (15)	N3—C18—C19—C24	−142.51 (12)
C6—N1—C7—C8	−174.73 (10)	O2—C18—C19—C20	−130.21 (13)
C10—N1—C7—C8	55.64 (13)	N3—C18—C19—C20	43.64 (17)
C9—N2—C8—C7	61.10 (14)	C24—C19—C20—C21	−0.53 (19)
C11—N2—C8—C7	−175.50 (11)	C18—C19—C20—C21	173.28 (11)
N1—C7—C8—N2	−58.92 (14)	C19—C20—C21—C22	2.09 (19)
C11—N2—C9—C10	175.83 (10)	C20—C21—C22—C23	−1.46 (19)
C8—N2—C9—C10	−61.23 (13)	C20—C21—C22—N5	177.83 (11)
C6—N1—C10—C9	173.32 (11)	O4—N5—C22—C21	−162.31 (12)
C7—N1—C10—C9	−56.22 (14)	O3—N5—C22—C21	17.24 (17)
N2—C9—C10—N1	59.47 (14)	O4—N5—C22—C23	17.01 (17)
C9—N2—C11—C12	−153.57 (10)	O3—N5—C22—C23	−163.44 (12)
C8—N2—C11—C12	85.39 (13)	C21—C22—C23—C24	−0.75 (19)
C18—N3—C12—C11	−81.95 (14)	N5—C22—C23—C24	179.97 (11)
C13—N3—C12—C11	79.80 (14)	C22—C23—C24—C19	2.35 (19)
N2—C11—C12—N3	−171.93 (10)	C20—C19—C24—C23	−1.74 (19)
C17—N4—C13—C14	2.34 (18)	C18—C19—C24—C23	−175.64 (11)

### Hydrogen-bond geometry (Å, °)

**Table:**

*D*—H···*A*	*D*—H	H···*A*	*D*···*A*	*D*—H···*A*
C10—H10A···O1	0.99	2.35	2.9536 (18)	119
